# Sinabro: A Smartphone-Integrated Opportunistic Electrocardiogram Monitoring System

**DOI:** 10.3390/s16030361

**Published:** 2016-03-11

**Authors:** Sungjun Kwon, Dongseok Lee, Jeehoon Kim, Youngki Lee, Seungwoo Kang, Sangwon Seo, Kwangsuk Park

**Affiliations:** 1 Interdisciplinary Program in Bioengineering, Graduate School, Seoul National University, Seoul 08826, Korea; sjkwon@bmsil.snu.ac.kr (S.K.); azuremoon@bmsil.snu.ac.kr (D.L.); jhkim119@bmsil.snu.ac.kr (J.K.); 2 School of Information Systems, Singapore Management University, Singapore 178902, Singapore; youngkilee@smu.edu.sg; 3 School of Computer Science and Engineering, KOREATECH, Cheonan, 31253, Korea; swkang@koreatech.ac.kr; 4 The 7th R&D Institute, Agency for Defense Development, Daejeon, 32024, Korea; prizeworthy3@gmail.com; 5 Department of Biomedical Engineering, College of Medicine, Seoul National University, Seoul 03080, Korea; pks@bmsil.snu.ac.kr

**Keywords:** opportunistic sensing, unobtrusive sensing, smartphone-integrated, phone case-type, ECG, sensor

## Abstract

In our preliminary study, we proposed a smartphone-integrated, unobtrusive electrocardiogram (ECG) monitoring system, Sinabro, which monitors a user’s ECG opportunistically during daily smartphone use without explicit user intervention. The proposed system also monitors ECG-derived features, such as heart rate (HR) and heart rate variability (HRV), to support the pervasive healthcare apps for smartphones based on the user’s high-level contexts, such as stress and affective state levels. In this study, we have extended the Sinabro system by: (1) upgrading the sensor device; (2) improving the feature extraction process; and (3) evaluating extensions of the system. We evaluated these extensions with a good set of algorithm parameters that were suggested based on empirical analyses. The results showed that the system could capture ECG reliably and extract highly accurate ECG-derived features with a reasonable rate of data drop during the user’s daily smartphone use.

## 1. Introduction

Daily electrocardiogram (ECG) monitoring provides pervasive healthcare applications with new opportunities. It enables heart diseases, such as arrhythmias, to be detected and can prevent sudden death caused by heart attack [1,2]. In addition, ECG can be utilized to infer stress level, emotion and sleep quality, since it is a sensitive indicator that shows changes in the autonomic nervous system (ANS) [1,3,4,5]. Daily ECG monitoring in various life situations is compelling, in order to bring these applications to reality.

Despite the high potential of daily ECG monitoring, its obtrusiveness makes it challenging for it to become widely deployed and available. Fundamentally, ECG sensing requires more than two body parts (with a sufficient electric potential difference) that are stably attached to the electrodes of a sensor. With this requirement, users of existing ECG monitoring systems need to intentionally touch the sensor electrodes continuously or wear uncomfortable wearable devices.

Prior studies have addressed such obtrusiveness. A popular approach is to reduce the user’s attention and consciousness by embedding the sensors into accessories, such as a wristband and a belt [2,3,6,7]. However, in this approach, monitoring is only possible when users are wearing such sensor-embedded accessories, limiting the use cases to clinical or fitness scenarios. Another interesting approach has been to integrate the sensing function with personal daily items, such as a smartphone. For instance, AliveCor [8] is a smartphone case equipped with an ECG sensor, enabling a user to measure ECG anywhere and anytime. However, AliveCor requires explicit initiation and attention from users. The user should consciously contact the sensor on the back of the case with the skin of the chest or hold the sensors with both hands to capture ECG. Such explicit attention being required makes consistent ECG monitoring challenging.

Our primary goal is to address this obtrusiveness by overlaying ECG monitoring onto daily smartphone use. For example, an ECG signal is opportunistically sensed without the user’s consciousness while the user is using his or her smartphone for calling, texting or gaming. This approach reduces the obtrusiveness by avoiding user intervention for sensing. Moreover, this approach enables diverse healthcare applications to be realized in everyday situations. For instance, a smartphone application can inform users of their stress levels or emotional status during phone calls and allow the users to respond appropriately, as they do naturally during face-to-face conversation.

While prior works have studied smartphone-integrated ECG sensing systems [2,3,6,8], opportunistic sensing approaches have hardly been considered. We need to overcome two main challenges in order to realize the opportunistic approach. First, we should investigate whether there are sufficient sensing opportunities during the user’s daily smartphone use to infer his/her health status. Second, we need to develop a technique to improve the reliability of the data that are opportunistically captured during the user’s natural smartphone use. The quality of the ECG signal captured by a smartphone-integrated sensor is easily reduced by various motion artifacts, e.g., playing an interactive game with continuous touching gestures. Such unreliable ECG data are likely to decrease the accuracy of inferring health status or even make it impossible.

In our preliminary study [9,10], we proposed an initial design for Sinabro and studied its performance. In this paper, we have extended the study’s scope by: (1) upgrading the sensor device; (2) improving the feature-extraction process; and (3) evaluating extensions of the system. First, we enhanced the design of Sinabro’s prototype sensor. The number of electrodes and their placement were changed to monitor ECG more reliably. On top of the raw ECG signal, Sinabro extracts diverse and reliable ECG-derived health features, such as heart rate (HR) and heart rate variability (HRV) parameters. For reliable feature extraction, we improved the extraction process, which intelligently removes noisy signals caused by motion artifacts and interpolates missing data, if possible. It also drops data with consecutively long, missed features to guarantee the reliability of the extracted features. We evaluated these extensions using a good set of algorithm parameters suggested based on empirical analyses.

## 2. Potential Opportunities for Daily ECG Monitoring

In the design of the Sinabro system, it is essential to understand whether sufficient opportunities to measure ECG signals during daily smartphone use exist. In this respect, we first review our previous study [9], in which we performed an experiment to answer the following questions:

- How many potential opportunities exist per day?
- How long does each opportunity last?
- How reliably can ECG signals be captured during the opportunities?

Fourteen participants (male/female: 10/4, age: 23–34) using an Android smartphone were recruited in this study. We collected their smartphone usage logs, which included a wide range of user interaction events, *i.e.*, start/end times of call events, touch events for typing, screen orientation change events and app use events, for six days, on average, using a custom-developed logging app. We analyzed the logs to figure out the number of ECG-sensing opportunities and their durations. The analysis focused on three smartphone use cases: (1) calling without using earphones, a headset or speakerphone; (2) text typing; and (3) gaming and taking pictures in landscape orientation. We targeted the smartphone use cases that included two hand contact or one hand and an ear contact with a smartphone. Users often hold a smartphone with two hands when typing on a keyboard, playing a game and taking a picture. During a phone call without accessories, users usually hold their smartphone with one hand and touch its head to one ear. The collected events were classified into five groups by time duration. The time durations were 10–30 s and 30–60 s for heart rate (HR) and ultra-short-term heart rate variability (HRV) analysis [5,11], 1–2 min for the high-frequency HRV components [1], 2–5 min for the low-frequency HRV components [1] and five or more minutes, which is the typically recommended duration for HRV analysis [1,4]. Note that the potential opportunities are not the opportunities when the ECG signal could actually be captured, but the potential feasibility for sensing.

**Figure 1:**
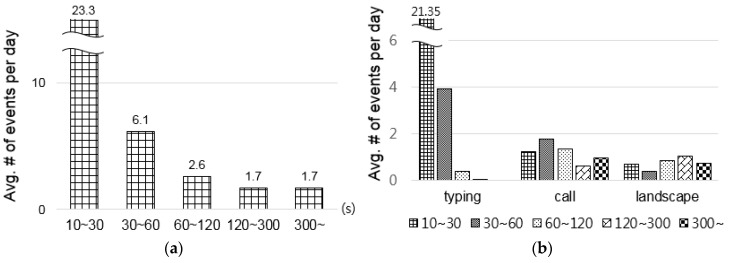
Average number of potential opportunities: (**a**) Different time durations; (**b**) different use cases.

The results showed that numerous potential opportunities existed for unobtrusive ECG monitoring throughout the day, as shown in Figure 1. The average number of potential opportunities varied from about 1.7–23.3, depending on the time durations (Figure 1a). Almost 30 opportunities existed for HR and ultra-short-term HRV analysis (10–30 s, 30–60 s) per day. Although the number of opportunities varied between individuals, the lowest number of potential opportunities was more than eight, and three users who used smartphones frequently showed more than 80 opportunities, on average. The average number of opportunities differed depending on the use case (see Figure 1b). The largest number of opportunities came out in the typing case. In the typing case, the number of opportunities with 10–30-second durations was remarkably larger than those of the other two cases, which might be opportunities for HR and ultra-short-term HRV analysis. The call and landscape cases had increased numbers of opportunities for larger time durations. Phone calls and gaming are likely to be continued for relatively longer; therefore, ECGs captured during those opportunities might enable HRV analyses lasting more than 2 min to be performed.

## 3. Sinabro Design and Prototype

Sinabro includes a phone case-type sensor and smartphone middleware. The sensor and the middleware work collaboratively to monitor reliable ECG-derived contexts and to deliver them to multiple pervasive healthcare applications through Sinabro APIs. As mentioned, we proposed the concept of an unobtrusive smartphone-integrated ECG monitoring system during natural smartphone use and an early prototype in our previous study [9]. In this study, we considerably extend our early work mainly in two aspects. First, we enhanced the sensor design of the Sinabro prototype for reliable ECG sensing. The number of electrodes and their placement were changed to capture ECG more reliably. The sensing hardware was also upgraded to capture multiple ECG channels at the same time. The simultaneously sensing of multiple ECG channels can feasibly enhance the quality of the captured signal using blind source separation techniques, such as independent component analysis (ICA) [12]. Second, we significantly improved the feature extraction process and developed a reliable QRS-peak-detection algorithm that intelligently removes noisy signals caused by motion artifacts and interpolates missing data, if possible. It also drops a long segment of consecutively-missed data from the feature extraction to guarantee the reliability of the extracted features. In the following subsections, we present the details of the new sensor design and the feature extraction process.

### 3.1. Phone-Case-Type ECG Sensor

**Figure 2:**
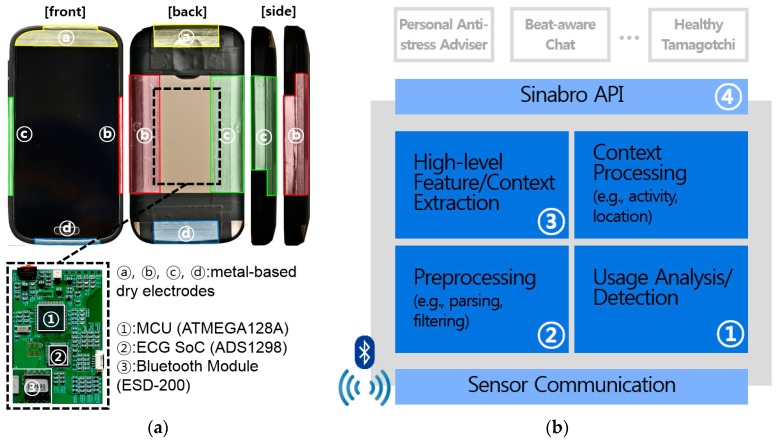
The prototype of the Sinabro sensor device and the architecture of the middleware: (**a**) the prototype of the phone case-type sensor; (**b**) the architecture of the Sinabro middleware.

We designed the Sinabro sensor device for reliable and durable ECG monitoring without user intervention during daily smartphone use. The new sensor prototype is shown in Figure 2. Multiple metal-based dry electrodes (Figure 2a, ⓐ-ⓓ) are placed on the outer frame of the case to come into contact with the user’s two hands during natural smartphone use. We also placed an electrode on the head of the front (Figure 2a, ⓐ-front) to sense ECG from the user’s ear during a call. The electrode ⓐ-front is the continuation of the electrode ⓐ. We used aluminum film for the electrodes, since it has enough conductivity to capture ECG and high durability against sweat and humidity. Furthermore, its thinness makes it easy to adapt to the curved surfaces of a smartphone. Our phone case-type sensor enables users to easily adapt the Sinabro system onto their existing smartphones.

**Figure 3:**
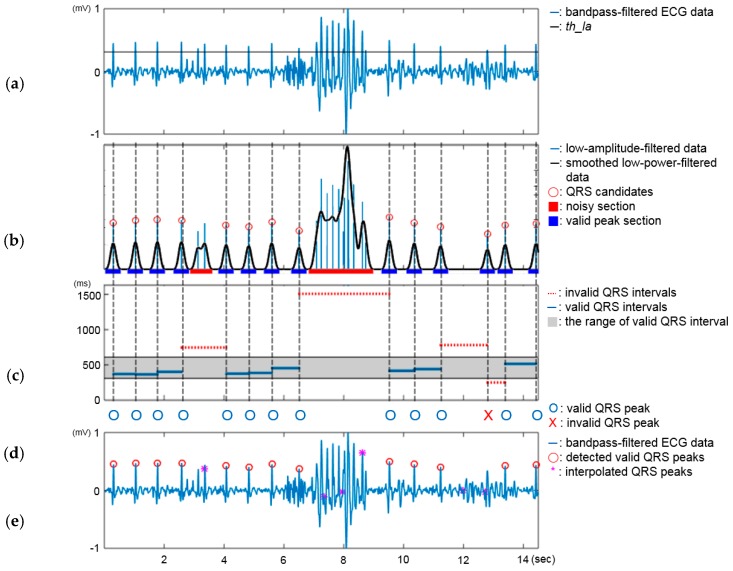
The detection and correction process of the QRS peak detector. (**a**) Blue solid line: bandpass-filtered ECG data; black solid line: the threshold for low-amplitude filtering, *th_la*; (**b**) blue line: low-amplitude-filtered data; black solid line: smoothed signal of the blue line; red circle: detected QRS candidates; red bar: noisy section; blue bar: valid QRS peak section; (**c**) gray box: the range of valid intervals; blue solid lines: valid intervals; red dotted lines: invalid intervals; (**d**) O: valid QRS peak; X: invalid QRS peak; (**e**) blue solid line: bandpass-filtered ECG; red circle: detected QRS peaks after correction; magenta star: interpolated QRS peaks.

The design of the prototype sensor mainly targets three use cases in daily smartphone use situations: phone calls, two hands in landscape orientation (e.g., during gaming, photo taking) and in portrait orientation (e.g., typing, gaming). During a phone call, the user’s ECG is captured from his/her ear and the hand that holds the smartphone by a combination of the electrode ⓐ-front and ⓑ or ⓒ. During the use cases with two hands in portrait orientation, ECG is captured by the electrodes ⓑ and ⓒ. In landscape orientation, the electrodes ⓐ-rear and ⓓ cover ECG sensing. As we mentioned in Section 1, ECG sensing fundamentally requires more than two body parts to be in contact with the sensor, which have a sufficient electric potential difference. With this requirement, holding the smartphone with one hand in portrait orientation was excluded in a target situation despite this quite frequent use case. The sensor device simultaneously monitors multiple ECG channels from four combinations of electrodes (ⓐ, ⓑ, ⓐ–ⓒ, ⓑ, ⓒ and ⓐ–ⓓ). We used a low-power integrated ECG front-end ADC (ADS1298, Texas Instruments, USA) (Figure 2a, ②) to perform major sensor-side processing. It extracts multiple ECG channels and converts the analog signals into 24-bit digital data. The digitalized multi-channel data are delivered to the micro controller unit (MCU) (ATMEGA128A, Atmel, USA) (Figure 2a, ①) via the serial peripheral interface (SPI), and the data are wirelessly forwarded to the smartphone through a Bluetooth interface (ESD-200, SENA, Korea) (Figure 2a, ③). The MCU parses the command from the middleware and controls the device, e.g., sensing turned on/off, configuring the sampling rate, *etc*. It also handles the Bluetooth module and ADC. To conserve energy consumption, the processing and communication modules on the sensor device are only turned on when the middleware detects a potential opportunity for ECG sensing. The default sampling frequency is 250 Hz, and it can be controlled as 125 Hz, 250 Hz or 500 Hz by Sinabro middleware in real time. There is a trade-off between the reliability of HRV parameters and the processing cost. A higher sampling rate reduces the error of RR intervals and it enables HRV parameters to be extracted more accurately. On the other hand, it requires more battery consumption and processing cost by increasing the data rate and wireless communication. Therefore, our system has an option to control the sampling rate at diverse resolutions depending on the purpose of the pervasive healthcare application using our system. The device consumes 166.5 mWh during processing and communicating and only 51 mWh in standby mode.

### 3.2. Sinabro Middleware for Extracting ECG-Derived Features

**Figure 4:**
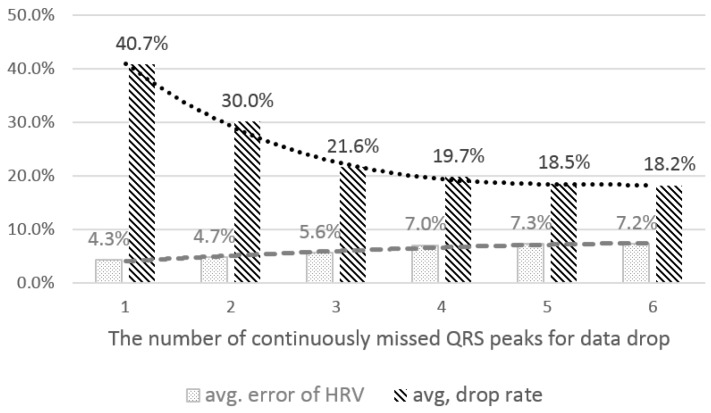
The average error of the HRV parameters and the average drop rate by the number of consecutively-missed QRS peaks.

The Sinabro middleware manages and controls the sensor device by monitoring the user’s usage. It extracts ECG-derived features from the captured ECG and delivers them to multiple pervasive healthcare applications. Figure 2b shows the architecture of the middleware. The middleware turns the sensor device on when a potential opportunity is detected and selects the most proper channel (a combination of electrodes) to sense ECG reliably by monitoring the user’s smartphone use (Figure 2b, ①). When the proper channel is selected, the middleware receives raw ECG data from the channel via Bluetooth connection and delivers them in real time to the preprocessor and the feature extraction module (Figure 2b, ②, ③). The raw ECG data and the extracted features can be delivered in real time to multiple applications using Sinabro APIs (Figure 2b, ④).

The overall operations for feature extraction are conducted as follows. First, the preprocessor of the middleware applies a digital bandpass filter (bandwidth: 7 Hz–35 Hz) to the raw ECG data, in order to reduce noise caused by environmental power, electromyogram (EMG) and hand motion. The applied cutoff frequency also helps to clarify QRS peaks in the signals. Then, the QRS peak detector intelligently filters out the noisy ECG data and finds reliable QRS peaks efficiently. It also includes a correction process for missed or wrongly-detected QRS peaks by detecting abnormal RR intervals. Finally, ECG-derived features (HR and HRV parameters) are calculated using RR intervals from the results of the QRS peak detector. To reduce the error of the extracted features in highly noisy situations, the QRS peak detector determines the data segments with many missing or incorrectly-detected peaks, which make it hard to perform accurate correction, and drops them from the extraction process. Note that the feature extraction process is applied to 30-s window data and is executed every second. The window size is compatible with ultra-short-term heart rate variability (HRV) analysis [5,11]. Other high-level features, such as stress level and affective state, can be further derived based on the extracted features [4,5].

#### 3.2.1. QRS Peak Detector

In many of the potential opportunities targeted by the Sinabro system, the users’ hands might interact with his or her smartphone continuously. Therefore, the captured signals can easily be exposed to the artifacts caused by the hand motion. It is difficult to detect QRS peaks correctly from ECG signals that are seriously distorted by artifacts. These wrongly-detected or missed QRS peaks can degrade the reliability of the QRS interval-based derived features, such as HR and HRV.

We developed a custom QRS peak detection algorithm that is suitable for the limited computing environment of smartphones to address the problem. It guarantees the reliability of the extracted features by dropping noisy data from the extraction process. In designing the algorithm, we prioritized the reliability of the features. Considering the results of our preliminary study (see Section 2), which showed the existence of numerous and frequent sensing opportunities in daily smartphone use, we thought that providing reliable ECG-derived features to healthcare apps was more important than providing more of them. We designed the algorithm to reconstruct missing or dropped ECG data, if possible, to increase the chances of feature extraction.

The processing flow of the algorithm is as follows (see Figure 3). First, the algorithm receives bandpass-filtered ECG data from the preprocessor and filters out the data with a lower amplitude than a low-amplitude threshold, *th_la* (Figure 3a). After the bandpass filtering, R waves are relatively clarified, so that their amplitude level becomes quite distinct from that of other waves, *i.e.*, P, Q, S, and T waves. Low-amplitude filtering applied to such ECG data can efficiently sort out QRS peak candidates and reduce the amount of data that the algorithm needs to manage and process. For example, assume that one-second windows of ECG data are collected at a sampling rate of 250 Hz and that the user’s heart rate is 60 BPM. Then, only one or two samples among 250 are a QRS peak, and the other samples are not. An important issue in this step is how to determine the threshold, *th_la*, since a more precise threshold can result in more accurate QRS peak detection. The threshold is obtained by a coefficient value times the mean of the QRS peak amplitudes. For the mean QRS peak amplitude, we considered that different users usually show different ECG features, such as QRS peak amplitude, and different use cases may also affect the signal power. Thus, we obtained the mean of the QRS peak amplitudes in a personalized way with calibration data, which are individually collected for 30 s in three use cases, *i.e.*, calling, holding in landscape orientation and holding in portrait orientation, while maintaining stable contact between the sensor and body through the user’s cooperation. Accordingly, we can set the personalized and case-sensitive thresholds for different users. The coefficient value was set as 0.6, which was determined based on our empirical analysis (see Section 4.2.).

Next, the algorithm detects noisy sections among the QRS peak candidates and filters them using a smoothing technique. When the smoothing technique is applied to the candidate data, the noisy data involve longer time durations than the clear QRS peaks (Figure 3b). Thus, the algorithm detects a noisy section whose width is larger than a certain threshold. The threshold is also empirically obtained; it is currently 1.05-times the mean width of the R wave from the smoothed calibration data. Through this step, most invalid QRS peak candidates can be removed.

The third step is a correction process for wrongly-detected or missed QRS peaks. This is important to improve the reliability of the derived features, such as HRV parameters. Wrongly-detected or missed QRS peaks degrade the accuracy of the derived features by inducing inaccurate RR intervals (Figure 3c). The correction process removes wrongly-detected QRS peaks mapped to abnormal intervals and recovers the missed QRS peaks using interpolation (Figure 3e). The details of the correction process are as follows. First, the algorithm detects invalid intervals. We statistically set the range of a valid interval length using the reference ECG data acquired for evaluation. We calculated mean and standard deviation (SD) of valid RR intervals in the reference data and set the range as mean RR interval ± 1.96 × SD. Next, the algorithm sequentially iterates all RR intervals to determine whether two detected peaks mapped to each of them are valid using the correction rule (shown in Table 1), which considers the conditions of the previous and later intervals. The head and tail peaks are defined as the front and rear peaks in two peaks mapped to the current interval, respectively. Thirteen intervals shown in Figure 3c were calculated from the detected peaks shown in Figure 3b. The correction results for the QRS peaks are shown in Figure 3d. The correction rule should be applied sequentially to the intervals. For example, when the correction process iterates the 11th interval, the current interval is invalid, so the tail peak is not fixed in this iteration. In the next iteration for the 12th interval, the current interval is invalid, and the head peak is not fixed; so the tail peak is not fixed, and the head peak is decided to be invalid.

After the correction process, the algorithm re-calculates the RR intervals from the corrected QRS peaks and finds the section where the QRS peaks are missing by detecting invalid long intervals. The algorithm interpolates the sections mapped to the invalid long intervals (Figure 3d) into multiple short intervals using a custom-developed interval split model [13] to recover the missed QRS peaks. The model predicts possible cases for the interpolation, including the estimated number and length of the short intervals. It uses the piecewise cubic Hermite (PCH) method for the interpolation. If the number of cases is greater than one, the model will choose the best case to be the one whose sum of the estimated lengths of the short intervals is most similar to the original length of the invalid long section.

The PCH method shows superior performance in interpolating missed QRS peaks [14]. However, if many QRS peaks are consecutively-missed, the performance of the interpolation would degrade rapidly. Therefore, the algorithm drops windows that include invalid long intervals with more than an estimated three missed QRS peaks. This threshold was also set based on the empirical analysis described in Section 4.2.

We conducted a simple test to measure the power consumption of Sinabro middleware in monitoring mode and standby mode using the “Battery” menu in the Android settings. According to the test, the power consumption of middleware in monitoring mode is 290.7 mWh. In standby mode, the “Battery” menu did not show the consumption because of its negligible amount.

#### 3.2.2. Sinabro APIs

Sinabro provides APIs to support diverse pervasive healthcare applications. Table 2 shows the key APIs of Sinabro. Two primitives, registerHRListener() and registerHRVListener(), enable applications to trace HR and HRV on the fly. Once an application is registered, it receives updates for the requested features from Sinabro. The application also assigns the target use cases using a condition argument, e.g., a phone call application monitors the user’s heart rate when the user is on the phone with a friend. Applications can also retrieve the user’s high-level contexts, such as stress and affective states, using registerContextListener(), which are derived from HR and HRV. The high-level contexts are derived using existing methods [4,5], and the methods are easily incorporated into our feature extraction logics. The applications also obtain historical data using getHistory(), e.g., “Let me know my HR and stress values when I was on a subway train to the office.” Sinabro provides a SQL interface to easily query stored information.

## 4. Evaluation

### 4.1. Experimental Setup

Fourteen participants were recruited from Seoul National University (male/female: 9/5; average age: 25.1; SD: 2.36) for this study. This study was approved by the Institutional Review Board of Seoul National University Hospital (IRB No. C-1512-110-728). We provided the participants with a smartphone equipped with the Sinabro prototype; we used the Samsung Galaxy S4 for the prototype. The sampling rate of the sensor device was set as 250 Hz. To collect raw ECG data and extract feature data from the Sinabro system, we asked them to use the smartphone naturally under five predetermined use cases: texting in the portrait and landscape orientations, playing highly and less interactive games in landscape orientation and calling without a hands-free device. Each use case was performed for 5 min.

The detailed settings for each use case were as follows. In the texting case, we asked the participants to have a conversation with the experimenter through a messenger app for following their natural typing. In the gaming case, they were asked to play two games with different interaction levels. One is a highly interactive action game, Touch Fighter for Kakao, which incurs frequent and strong inputs (at least 1–2 inputs per second, up to five). The other was the low-interaction baseball game, Com2uS Professional Baseball 2015, which involves relatively less frequent, gentler inputs (avg. 0.5 per second). Both games were played with two hands in landscape mode. For calling, the participants were asked to have a conversation with the experimenter through a phone. They held the phone with their left hand and touched it to their left ear.

During the use cases, the raw ECG data sensed from a channel selected by the Sinabro middleware were collected. The detected QRS peaks and derived features by the middleware were also obtained. The reference ECG data were simultaneously collected using a conventional ECG acquisition system, BIOPAC MP150 with the ECG100C module [15], with Ag/AgCl electrodes, which were attached to the left and right forearms.

Before the experiment, we collected raw ECG data for 30 s under stable contact with the sensors’ electrodes as the personal calibration data. The calibration data were collected for three cases: holding in the portrait and landscape orientations and calling without any motion in the contacted body parts.

### 4.2. Parameter Setup in the QRS Peak Detector

In this subsection, we present empirical analyses to determine the parameter values used in QRS peak detection. As mentioned, the QRS peak detector uses multiple thresholds to filter out unreliable ECG data before feature extraction, in order to provide accurate peak detection and reliable feature extraction, e.g., the threshold for low-amplitude filtering, detecting noisy sections in smoothed data of QRS peak candidates and dropping data with long, consecutively-missed QRS peaks. These thresholds can make a trade-off between the reliability of the extracted features and the rates of the dropped or filtered data. Precisely-refined thresholds allow us to avoid incorrect filtering and to keep the reliability of the extracted features at a reasonable level. Before the system evaluation, we performed three analyses to find better thresholds, in order to decrease the error of the extracted features and the rates of the dropped data at the same time. We used the average error of the HRV parameters and the average rates of the dropped data as the evaluation metrics for the analyses. The parameters are described in Section 4.3.

#### 4.2.1. The Number of Consecutively-Missed QRS Peaks for Data Drop

First, we looked into the desirable number of consecutively-missed QRS peaks to decide whether a window of data should be dropped. If the number is large, we can include many data windows for feature extraction. However, the error of the extracted features, such as the HRV parameters, can increase, since there are many consecutively-missed peaks, which make it difficult to correctly interpolate the missed peaks. On the other hand, if the number is small, we might drop many data windows, and thus, the chance to extract the features can decrease. However, the reliability of the extracted features from the remaining windows would be improved. We calculated the average error of HRV parameters and the average drop rates of the data windows under different thresholds, *i.e.*, 1~6. We controlled the coefficient value for the low-amplitude filtering threshold as 0.6 and used personalized and case-sensitive thresholds for this analysis.

The results show the trade-off between the average error of HRV and the drop rate by the threshold (see Figure 4). In the threshold from 1–3, the drop rate decreased rapidly, but the error slightly increased. From 3–6, the trends of increasing error and decreasing drop rate are similar. Based on the results, we empirically set the threshold as three for data drop.

#### 4.2.2. The Effect of the Personalized, Case-Sensitive Threshold

Next, we examined the effect of a threshold for low-amplitude filtering. As shown in Figure 3a, the QRS peak detector first applies low-amplitude filtering to select QRS peak candidates. A key parameter for determining the threshold, *th_la*, is the mean of the QRS peaks’ amplitudes in the calibration data. QRS peaks usually show different amplitudes, depending on the user. In addition, since Sinabro captures ECG signals under different use cases, the amplitudes of the captured QRS peaks can be different, even for the same user. This is mainly because the size of the electrodes contacting the body and the body parts differs in different use cases. For example, a user’s hand posture in landscape orientation might be different from that in portrait orientation, and the body parts contacting the sensors while playing a game are different from those when calling. We investigated the effect of a personalized and case-sensitive threshold on the performance of the feature extraction to set the thresholds, by considering individually different ECG features and different sensing conditions in different use cases. We compared the evaluation metrics from the calibrated thresholds with those from the unified threshold. While the unified threshold was set as the mean peak amplitude from the calibration data of all of the participants in all of the use cases, the personalized threshold was set as the mean peak amplitude from the personal calibration data. The case-sensitive threshold was set as the mean peak amplitude from the calibration data of all of the participants in the target use case. The number of consecutively-missed QRS peaks for data drop and the coefficient value for low-amplitude filtering were controlled as three and 0.6, respectively.

**Figure 5:**
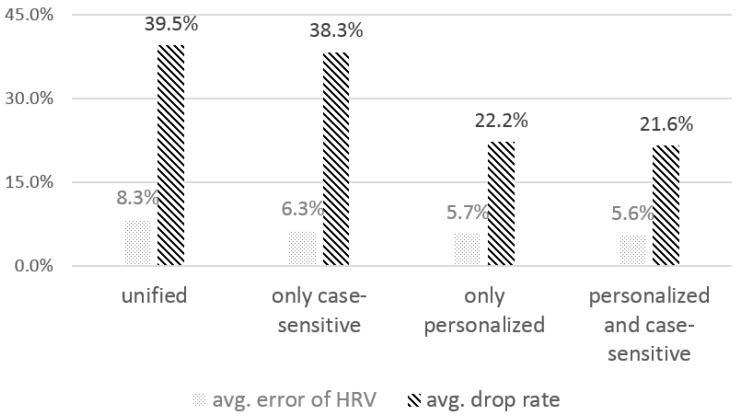
The effect of the personalized and case-sensitive threshold in noise filtering on the average error of the HRV parameters and the average drop rate.

Figure 5 shows the analysis results. When the unified threshold was used, the average error and the drop rate were 8.3% and 39.5%, respectively, which were the highest among all of the thresholds. When applying the case-sensitive threshold, the HRV error decreased by 2%, and the drop rate also decreased slightly, by 1.2%. With a personalized threshold, the drop rate decreased significantly to 22.2%, and the HRV error also decreased by 2.6%. The smallest HRV error and drop rate were obtained when a personalized and case-sensitive threshold was applied. The results show that the personalized and case-sensitive threshold filtered out the noise signals more precisely than the unified threshold. While the personalized and case-sensitive threshold has the advantage of better performance, personal calibration data need to be collected from users in order to apply the personalized and case-sensitive threshold. Since the calibration data collection would be a one-time operation and the time for it is reasonably short, about 30 s for each of the target use cases, it would only be a minor burden for users.

#### 4.2.3. The Coefficient Value to Derive the Threshold for Low-Amplitude Filtering

We analyzed the performance of the feature extraction with different coefficient values to set the refined value of the threshold, *th_la*, for the low-amplitude filtering. We calculated the evaluation metrics by changing the coefficient value from 0.3 to 0.8. For this analysis, we set the number of consecutively-missed QRS peaks for dropping data as three and used a personalized and case-sensitive threshold.

**Figure 6:**
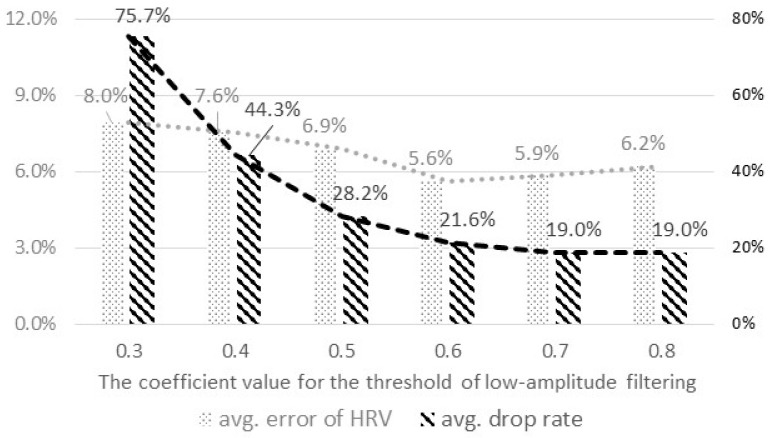
The average error of the HRV parameters and the average drop rate by the coefficient values for the threshold of low-amplitude filtering.

Figure 6 shows the results of the analysis. The drop rate greatly decreased by 54%, with the coefficient value increasing from 0.3 to 0.6, and it was saturated at 0.6. The average error decreased from the range of 0.3–0.6 and increased again after 0.6. Based on the results, we set the coefficient value of the threshold for low-amplitude filtering as 0.6, which showed the smallest error, as well as a low drop rate.

### 4.3. Sensing and Feature Extraction in Actual Opportunities

We evaluated three aspects of the Sinabro prototype. First, we investigated how reliably the Sinabro sensor device can capture ECG signals in actual sensing opportunities. Second, we evaluated the accuracy of QRS peak detection. Third, we examined the performance of the feature extraction. We assumed that the actual sensing opportunity is the moment when the users interact with their smartphones using two hands or when they are on the phone without using an earphone or hands-free device.

The evaluation metrics were as follows. First, we used the QRS peak detection ratio (PDR) to evaluate the sensing reliability of the Sinabro device. The PDR is defined as the ratio of the number of QRS peaks that experienced experts could manually detect from pre-filtered ECG data provided by Sinabro to the number of the QRS peaks detected from the reference ECG data. Because the experts could correctly detect more QRS peaks from clearer ECG data, the PDR shows the reliability of the ECG data sensed by the Sinabro device.

Second, we used sensitivity (*Se*), the positive predictive value (*PPV*) and error (*Er*) as evaluation metrics to evaluate the accuracy of the QRS peak detection algorithm. This is important, since the accuracy of the QRS peak detection is a key factor to reliably derive the RR interval-based features. The definitions of the metrics are given in Equation (1). TP, FP and FN were calculated by comparing the QRS peaks detected by the peak detection algorithm with the QRS peaks that the experienced expert detected manually from the reference ECG data.

(1) $$Se=\frac{\text{TP}}{\text{TP}+\text{FN}}\times 100\left(\%\right),\;PPV=\frac{\text{TP}}{\text{TP}+\text{FP}}\times 100\left(\%\right),\;Er=\frac{\text{FP}+\text{FN}}{\text{TP}+\text{FN}}\times 100\left(\%\right),\;\begin{array}{c} \text{TP: the number of valid QRS peaks correctly detected by the algorithm}\\ \text{FP: the number of QRS peaks wrongly detected as valid peaks by the algorithm}\\ \text{FN: the number of valid QRS peaks not detected by the algorithm} \end{array}$$

*Se* is how many real QRS peaks were correctly detected or interpolated by the algorithm. *PPV* is how many peaks that were detected as valid were real QRS peaks. *Er* is the ratio of the missed and wrongly-detected peaks to the real QRS peaks.

Third, we calculated the average error rate of the derived features, including the HR and HRV parameters, to evaluate the feature extraction performance. The error rate was obtained by comparing the HRV parameters derived from the QRS peaks provided by Sinabro with those from the reference ECG data. We also investigated the linear relationship between the HRV parameters from Sinabro and those from the reference data using the Pearson correlation.

Feature extraction was performed every second with 30-s sliding time windows. The coefficient value of the threshold for the low-amplitude filtering was 0.6, and we set personalized and case-sensitive thresholds using the calibration data. The number of consecutively missed QRS peaks for the data drop threshold was set as three. The extracted HRV parameters included the time-domain parameters, mean HR, SDNN and RMSSD, frequency-domain parameters, LF, HF, normalized HF (nHF), normalized LF (nLF), TF and LF/HF [1]. SDNN is SD of all normal RR intervals, and RMSSD is the square root of the mean of the squares of the successive differences between adjacent RR intervals. LF and HF are the power distribution across the low frequency from 0.04 HZ to 0.15 Hz and high frequency from 0.15 HZ to 0.4 Hz, respectively. TF is total spectral power under 0.4 Hz. Because of the limitation of the size of time windows, the extracted parameters do not include VLF, which is the power distribution across the very low frequency from 0.0033 HZ to 0.04 Hz. LF/HF is the ratio calculated by dividing LF power by HF power.

#### 4.3.1. Sensing Reliability

**Figure 7:**
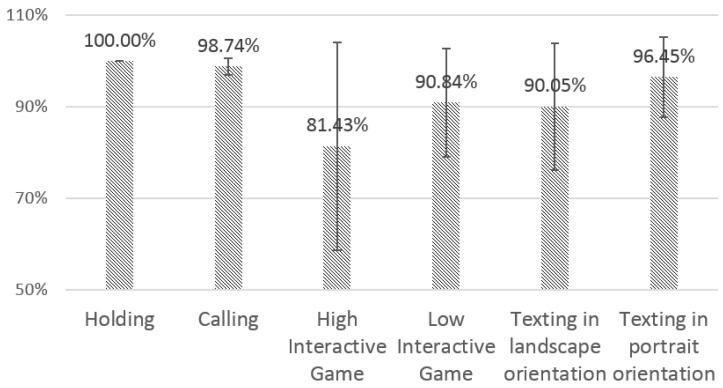
Average QRS peak detection ratios (PDRs) and SD for different use cases.

Figure 7 shows the results of PDR. There were no missed QRS peaks in the 30-second holding cases. This shows that our device can capture clear ECG signals under the condition of stable contact between the sensor and the hands. For texting in portrait orientation and calling, most of the QRS peaks were correctly detected; the PDRs were 98.7% and 96.5%, on average, respectively. In contrast, there were relatively more missed peaks in the other three cases, *i.e.*, texting and playing high- and low-interaction games in landscape orientation. The average PDRs varied from 81.4% to 90.8%, and their SDs were relatively large (11.8%–22.6%). We observed that four participants among the fourteen showed quite low PDRs in the case of highly interactive gaming (33.2%–66.9%). While playing the low-interactive game and texting in landscape orientation, two and three participants, respectively, also showed much lower PDRs (54.8%–77.5%). Except for those participants, the average PDRs were larger than 96%.

#### 4.3.2. Performance of the QRS Peak Detector

The QRS peak detector precisely detected or corrected almost all of the QRS peaks in reliable ECG data. *Se* was 99.9% on average, and only 0.1% of real QRS peaks were not detected. *PPV* was also very high, and *Er* was very low: 99.6% and 0.5%, respectively. Only 0.4% of the peaks detected by Sinabro were not correct QRS peaks. The results did not show significant differences among the different use cases. Even in the case of highly interactive gaming, for which the algorithm showed the worst results, the QRS peak detector missed only 0.2% of the real QRS peaks, and only 0.5% of the detected peaks were wrong. However, about 38% of the data were dropped in that case. Note that *Se*, *PPV* and *Er* were calculated by counting the matched peaks in the results from the QRS peak detector with the real QRS peaks in the reference ECG data, which were manually detected by the experienced experts. Therefore, these results do not include the accuracy of the RR intervals calculated from the results of the QRS peak detector.

The QRS peak detector dropped an average of 21.6% of the data from the feature-extraction process. Relatively more data were dropped in the use cases in landscape orientation. Those use cases showed an average 32.3% drop rate. Participants 3, 7, 9 and 14 showed higher average drop rates than the others in the use cases. Except for the results of these four participants, the average drop rate was decreased to 8.1%. For calling and texting in portrait orientation, only 3.9% and 8.7% of the data were dropped on average, respectively.

#### 4.3.3. Performance of the Feature Extraction

**Figure 8:**
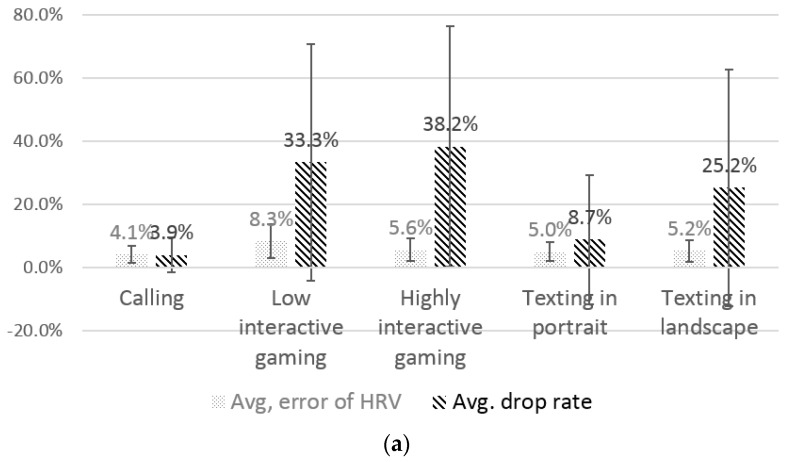

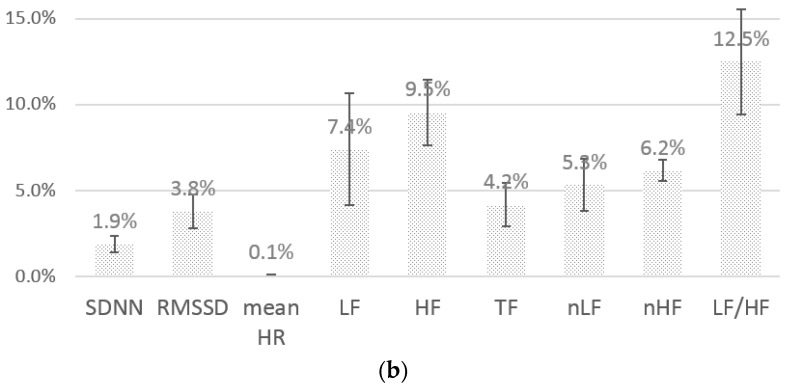
The average error and SDs of the HRV parameters. (**a**) Different use cases; (**b**) different parameters. SDNN, SD of all normal RR intervals; RMSSD, square root of the mean of the squares of the successive differences between adjacent RR intervals; LF, low frequency; HF, high frequency; TF, total spectral power; nLF and nHF, normalized LF and HF.

The results showed that the Sinabro system can feasibly provide highly reliable ECG-derived features. Figure 8a shows the average error of the HRV parameters and the average drop rates for the different use cases. Overall, the average error of the HRV parameters was only 5.6%. The error in the low-interaction gaming was slightly high, 8.3%, while the errors of the other use cases were less than 5.6%. The use case of low-interaction gaming showed a different noise pattern that was short and had a similar amplitude as the QRS peaks, but frequently appeared between QRS peaks. Therefore, much of the data in that case were filtered by the smoothing technique, but not dropped, because of its shortness. This means that many of the data’s QRS peaks were interpolated by the correction process, which increased the error. In the use case of highly-interactive gaming, once the noise occurred, it was relatively long and had a high amplitude. Therefore, its drop rate was about 5% higher than the low-interaction gaming, even though its error was slightly lower.

In the average errors of different parameters (see Figure 8b), most of the parameters showed less than an average of 10% error, except LF/HF. The time-domain parameters SDNN, RMSSD and mean HR showed relatively low error. The average error of SDNN was only 1.9%. The average error of mean HR was especially low, at only 0.1%, which is almost completely accurate heart rate estimation. In the frequency-domain parameters, few parameters showed somewhat noticeable errors. The average errors of LF and HF showed relatively high error rates, and they caused the high error of LF/HF. They were sensitive to the interval errors from the interpolated QRS peaks. However, the other parameters, TF, nLF and nHF, showed less than 5.3% average error.

Sinabro also showed high feasibility in providing reliable variation trends of HRV parameters. The HRV parameters extracted by Sinabro showed significantly high Pearson correlations with those from the reference data (see Table 3). The average correlation was 0.97, and the SD was only 0.04. We calculated the average correlation using only the correlations whose *p*-values were less than 0.01 to guarantee that the results would be statistically significant. Most of the data showed *p*-values lower than 0.01. The ratio of the low *p*-value data was an average of 97%. The errors of LF, HF and LF/HF were relatively high: greater than 5%. However, they showed a very significant linear relationship (a correlation greater than 0.95) with the reference data. Therefore, Sinabro has an advantage in supporting pervasive healthcare applications with an interest in the variation trends of HRV parameters.

## 5. Discussion and Future Work

Unobtrusiveness is a key factor to turn daily physiological sensing into reality. Attaching additional sensors onto the body and carrying a separate device for monitoring are possible solutions. However, users often feel that carrying more devices and initiating manual measurements are cumbersome, making daily monitoring challenging. On the other hand, we are inspired by the increasing adoption of metal bands in commercial smartphone designs (e.g., iPhone series, Vega IRON). Sinabro opportunistically senses ECG signals during daily smartphone use using a phone case-type sensor, reducing the users’ burden for manual sensing significantly.

The study in the computer–human interface (CHI) community will help Sinabro detect potential opportunities more accurately. There is a rich body of work on recognizing smartphone use activities in the CHI community. For instance, Kim *et al.* [16] proposed a system to recognize hand grip patterns when using mobile devices. They covered an array of capacitive touch sensors on mobile devices and estimated the user’s hand grip by monitoring the activated touch sensors. Goel *et al.* [17] proposed a system to detect the hand posture and pressure on a smartphone. The system infers the user’s hand posture by recognizing the rotation of the device, the strength of touch and the shape of the swipe arc using built-in sensors. The system detects diverse hand postures, such as one-hand grip during smartphone use. Sinabro can improve its reliability and save processing costs by accurately detecting potential opportunities based on such prior techniques.

**Figure 9:**
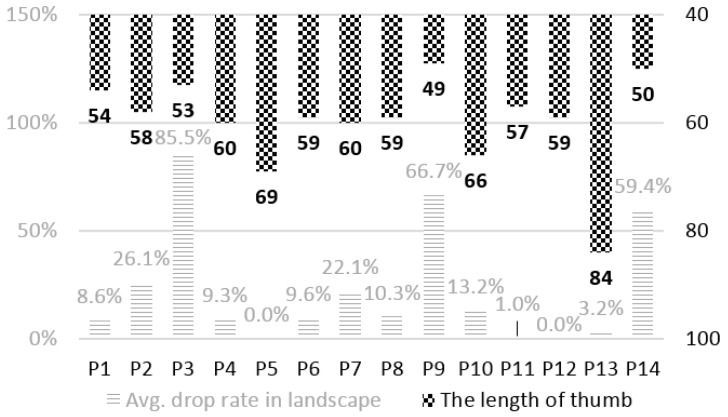
The average drop rate and thumb length of the participants.

We have several interesting findings beyond those reported earlier, which could lead to improved performance for Sinabro. For instance, the drop rates during the landscape-mode use of the phone showed a high correlation with the participant’s thumb length. Figure 9 shows the average drop rates of the participants and their thumb length. The overall average thumb length of the participants was 59.8 mm (SD: ±8.5). The thumb lengths of three participants—3, 9 and 14—who showed relatively large drop rates were 53, 49, and 50 mm, respectively. They had shorter thumbs than the others. According to the Pearson correlation, the correlation coefficient was −0.58, and the *p*-value was 0.03 (<0.05). Currently, many commercial smartphones are equipped with big screens with vertical sizes above 11 cm. When using a smartphone with both hands in the landscape mode, users often touch the screen using their thumbs. Users with short thumbs are likely to change their hand posture (more than those with longer thumbs) when they touch the center area of the screen. Such changes in hand posture cause significant motion artifacts and reduce sensing accuracy by temporarily separating the hands and electrodes.

## 6. Conclusions

In this paper, we first introduced an unobtrusive smartphone-integrated ECG monitoring system, Sinabro, which monitors a user’s ECG opportunistically during daily smartphone use without explicit user intervention. We extended the proposed system by: (1) upgrading the sensor device; (2) improving the feature extraction process; and (3) evaluating extensions of the system. We conducted experiments to evaluate the fully-functioning Sinabro system. The results showed that the sensor device could capture ECG reliably in target situations and that the middleware could extract highly accurate HRV features with a reasonable rate of data drop. We are planning further studies to retrieve users’ high-level contexts, such as stress and affective state level features, on the Sinabro system and will develop pervasive smartphone healthcare apps based on our system.

## Figures and Tables

**Table 1:** The correction rule for the wrongly-detected QRS peaks by invalid short intervals.

Current Interval	Head Peak	Tail Peak
valid	valid	valid
	invalid	valid
	not fixed → valid	valid
invalid	valid	not fixed
	invalid	not fixed
	not fixed → invalid	not fixed

**Table 2:** Key APIs. HRV, heart rate variability.

Monitoring HR and HRV	registerHRListener(callback(HR), condition) registerHRVListener(callback(HRV), condition) * condition = TARGET_APP|TARGET_MODE class HR {long timestamp; int HR;} class HRV {long timestamp; float LF; float HF; float LF/HF; float RMSSD; float SDNN; …};
Monitoring HR-/HRV-derived contexts	registerContextListener(callback(Context), condition, type) * type = STRESS|AFFECTIVE_STATE|…

**Table 3:** The average correlation, SD and ratio of the correlations whose *p*-values are less than 0.01.

	SDNN	RMSSD	Mean HR	LF	HF	TF	nLF	nHF	LF/HF	Average
avg. correlation	0.99	0.95	1.00	0.98	0.95	0.98	0.98	0.98	0.96	0.97
SD	0.05	0.09	0.01	0.06	0.06	0.05	0.02	0.02	0.03	0.04
ratio, *p* < 0.01	96.9%	96.9%	98.4%	96.9%	95.3%	98.4%	96.9%	96.9%	96.9%	97.0%
